# Changes in Pain and Nutritional Intake Modulate Ultra-Running Performance: A Case Report

**DOI:** 10.3390/sports6040111

**Published:** 2018-10-04

**Authors:** Russ Best, Benjamin Barwick, Alice Best, Nicolas Berger, Claire Harrison, Matthew Wright, Julie Sparrow

**Affiliations:** 1 Centre for Sport Science & Human Performance, Waikato Institute of Technology, Hamilton 3288, New Zealand; N.Berger@tees.ac.uk; 2 School of Health and Social Care, Teesside University, Middlesbrough TS3 1BX, UK; J.Sparrow@tees.ac.uk; 3 Full Potential Performance Ltd., Plymouth PL4 0RA, UK; ben@fullpotential.co.uk; alicebackhurst@me.com; 4 Freelance Dietician, Newcastle upon Tyne NE1, UK claire@c-harrison.co.uk; 5 Teesside Sport, Teesside University, Middlesbrough TS3 1BX, UK; M.Wright@tees.ac.uk

**Keywords:** ultra-endurance, running, nutrition

## Abstract

Ultra-endurance running provides numerous physiological, psychological, and nutritional challenges to the athlete and supporting practitioners. We describe the changes in physiological status, psychological condition, and nutritional intake over the course of two 100-mile running races, with differing outcomes: non-completion and completion. Athlete perception of pain, freshness, and motivation differed between events, independent of rating of perceived exertion. Our data suggest that the integration of multiple sensations (freshness, motivation, hunger, pain, and thirst) produce performance. Increases in carbohydrate feeding (+5 g·h^−1^) and protein intake (+0.3 g·kg^−1^) also likely contributed to successful completion of a 100-mile race, by reducing the fractional utilization of maximal oxygen uptake and satiating hunger, respectively. Nutritional data support the notion that the gut is a trainable, and critical organ with respect to ultra-endurance performance. Finally, we propose future research to investigate the rate at which peak feeding occurs throughout ultra-endurance events, as this may further serve to personalize sports nutrition strategies.

## 1. Introduction

Ultra-running is considered to be any running event in excess of a standard marathon (42.2 km [1]). Participation in these events has increased in recent years, on a domestic (Great Britain) and European scale, independent of age or gender [2]. Races are staged in either single-day or multi-day formats, with the latter offering the opportunity for overnight recovery between race segments. Other events have standardised race distances (50 km, 50 miles, 100 km, and 100 miles [1]), or time periods (6 h, 24 h, and multi-day events [1]). Differing terrain and environmental conditions, including extreme temperatures or high altitude, affect the difficulty of such events.

Throughout ultra-marathons interactions between psychological and physiological factors can influence performance [3], yet currently these are poorly understood [3]. These interactions may hold a key to better understanding what limits performance in this group of athletes [4], especially in amateur participants [3,5]. Athletes’ rating of perceived exertion (RPE) may oscillate during an event in accordance with fluctuations in other characteristics, such as pacing, pain [4,6], mental fatigue [7], and maximal sustainable power [5]. It is unlikely that these factors act in isolation with respect to performance. More likely, an athlete’s psychological state interacts with their physiological condition, and the environmental conditions, with all fluctuating throughout the race [8,9].

Many physiological variables affect ultra-marathon running performance, most notably the energetic cost of running (running economy) and anthropometric characteristics of an individual [10] with lactate threshold (LT) and maximal oxygen uptake (VO_2max_) also being considered physiologically important [11]. In comparison to shorter distances, a lower fractional utilisation of VO_2max_ must be sustained over a much longer duration, although this has been demonstrated to increase throughout a 90 km ultra-marathon [12], incurring a progressive worsening of the cost of running. Ultra-endurance activity also places significant demands upon the muscular and osteo-articular systems, with substantial changes in bone health [4,13] and bone metabolism [14] being reported. Successful adaptation, and not deterioration of muscular and osteo-articular systems might be a contributing factor to race success [11].

Appropriate nutrition [15,16,17] and hydration [18,19] strategies may attenuate performance decrements during ultra-endurance running. Meeting energy requirements is challenging [20,21] and achieving adequate carbohydrate (CHO) intake is preferable to maximise overall calorie consumption [15,20,21]. A sustained and well-tolerated CHO intake, preferably from multiple transportable sources [22], has been shown to support prolonged high-intensity activity, with more pronounced effects being observed in longer events [23]. Gastro-intestinal (GI) symptoms during ultra-endurance events are frequently reported; therefore, attenuation of GI symptoms either through nutritional training [24] or physiological adaptation [22] might play a part in successful completion and competitive racing over ultra-distances. Carbohydrate intakes of 71 ± 20 g per hour have been reported in elite ultra-marathon runners [17], attaining current recommendations [22].

This case study describes the physiological and psychological determinants of ultra-endurance running performance, over two contrasting attempts (non-completion vs. completion). The inclusion of nutritional data adds to the small body of empirical evidence currently available on this aspect of ultra-endurance performance.

## 2. Materials and Methods

The participant’s characteristics are presented in Table 1. The participant had 5.5 years running experience and it had recorded a marathon personal best of 2:56:49 as part of their preparation for the first recorded attempt at an ultra-endurance event (100 miles, non-completion). Mean weekly training volume throughout this period was 60 km·week^−1^, and it consisted of a variety of training sessions including long runs, interval training, continuous easy running, and conditioning work as prescribed and monitored by their coach. The participant had one unsuccessful ultra-distance attempt prior to seeking support. In between the attempts that are reported in this article, the participant completed a multi-stage ultra-running event, with a mean running distance of 46.km·day^−1^ over five days.

Ethical approval was obtained from the Teesside University ethics committee, with written informed consent from the participant, in accordance with the Declaration of Helsinki. Data were collected at the start and finish of each event, and at official checkpoints throughout. A researcher walked outwards from each checkpoint to meet the participant, and record subjective measures as the participant walked in to the checkpoint where the physiological measures were taken. Finally, the participant’s nutritional intake would be recorded, and beverages, foodstuffs, or sports nutrition products would be afforded to the participant before continuing with the race(s). Data collection took ~90 s per checkpoint during non-completion, and ~60 s per checkpoint during completion.

Blood glucose and blood lactate values were obtained through finger prick blood sampling, and recorded using portable analysers (Wireless Smart-Gluco Monitoring System, iHealth Labs, Mountain View, CA, USA; Lactate Pro, Cycle Classic Imports, Adelaide, Australia, respectively). All of the blood samples and sampling equipment were disposed of safely, and subsequently incinerated following analysis. The participant’s bodyweight (kg) was recorded at each checkpoint while using portable scales (Seca 803, Seca, Birmingham, UK), and change in bodyweight (Δ BW) calculated as the difference between the current and previous checkpoints’ values. Hydration status was self-reported via subjective urine colour score, using a previously validated eight-point scale [25]. In the incidence of no urine output, a score of 0 was noted.

Blood glucose and lactate sampling were not included for the second attempt (completion). Data for both measures were consistent throughout the course of the unsuccessful attempt, with little range in values being obtained. RPE was thought to be a sufficient reflection of exercise intensity and provision of a similar feeding strategy would elicit similar post-prandial blood glucose values. The exclusion of these measures from the battery also sped up data collection by ~30 s per checkpoint, thereby reducing time deficits incurred because of data collection.

Visual analogue scales (VAS; 100 mm) were used to record feelings of freshness, motivation, hunger, thirst, and pain. Freshness was defined as ‘readiness to continue’, motivation as ‘willingness to continue’ and pain as ‘an unpleasant sensory and emotional experience associated with actual or potential tissue damage’ [26]. Hunger and thirst are homeostatic processes, and therefore were not defined. Each scale represented a continuum from the worst possible outcome, to the best possible outcome, for each sensation. The participant was provided with an example scale on each data collection sheet (one sheet per crewed checkpoint) to ensure accurate and consistent interpretation of the VAS. The participant placed a vertical line at a point along the scale for each characteristic, at each crewed checkpoint, to represent their feeling of each sensation. These data were used to inform nutrition recommendations that were made during the event. Rating of perceived exertion (RPE) was recorded while using a 10-point scale (CR-10; [27]).

Nutritional intake was recorded using a semi-quantitative approach. The number of sports nutrition products consumed by the athlete between checkpoints was tallied, and then the athlete was questioned about other foodstuffs and beverages that they had consumed at non-crewed aid stations. These items were either freely chosen from un-crewed checkpoints manned by race support staff, or given to the athlete directly by the crew. Intakes of foods, liquids, and sports nutrition products were collated and analysed using specialist software (Nutritics Ltd. Co., Dublin, Ireland). Absolute (g) and relative (g·kg^−1^) carbohydrate and protein consumption were calculated, with total energy intake (kcal) also being reported and principal carbohydrate sources presented separately (glucose, fructose, and sucrose).

Raw data were inputted into Microsoft Excel (Microsoft Corporation, Redmond Washington, WA, USA) for analysis. Pearson’s correlations were calculated to assess the association between subjective measures that were reported on the 100 mm VAS scale across each race. The uncertainty in these data were reported via 90% confidence intervals and the magnitude of the correlation coefficient was attributed to qualitative descriptors [28]. The following correlation coefficients and descriptors were used: 0.0–0.1 Trivial; 0.1–0.3 Small; 0.3–0.5 Moderate; 0.5–0.7 Large; 0.7–0.9 Very Large; and, 0.9–0.1 Near Perfect.

We assessed the trends across each race using a customised spread sheet for the analysis of individuals, considering both the value that represented the minimal important difference and the typical error of the measure [29]. We derived the slope and its standard error and apply these data to understand the likelihood of the athlete experience a substantial decline in subjective wellbeing or increase in RPE at 10, 20, 40, 60, and 80 miles. We were also able to estimate the point at which the athlete was “likely” to demonstrate this substantial decline in each race, as well as any “likely” substantial changes between check points. Here, likely was defined as a 75% chance of a substantial negative change [28]. Between race differences were analysed using a separate customised spreadsheet [30]. The uncertainty in the data was reported via 90% confidence intervals and qualitative inferences were applied as per recommendations [28,31].

A typical error for our RPE data was taken from previous literature in endurance athletes using the CR-10 scale, 0.69 arbitrary units [32], and the minimal important change was set at 1 arbitrary unit as this can equate to an important difference between verbal anchors e.g., moderate to somewhat hard. We identified that the minimal important change in VAS data as 12 mm and applied this to all measures of freshness, motivation, hunger, thirst, and pain. This is the mean change that is associated with a patient feeling their pain is “a little bit worse” [33]. A typical error of 9 mm was chosen for VAS scale measures [34].

## 3. Results

### 3.1. Physiological and Subjective Measures

**Figure 1:**
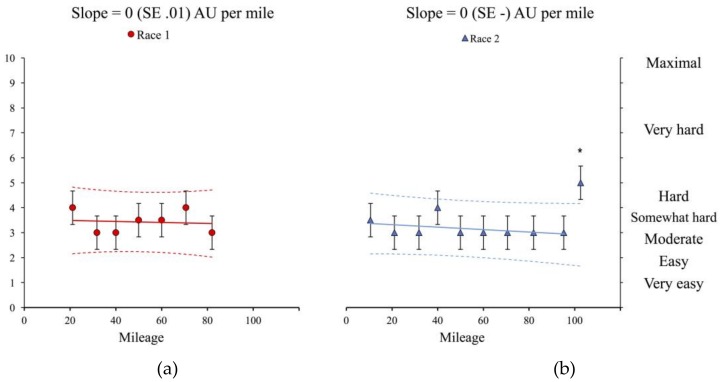

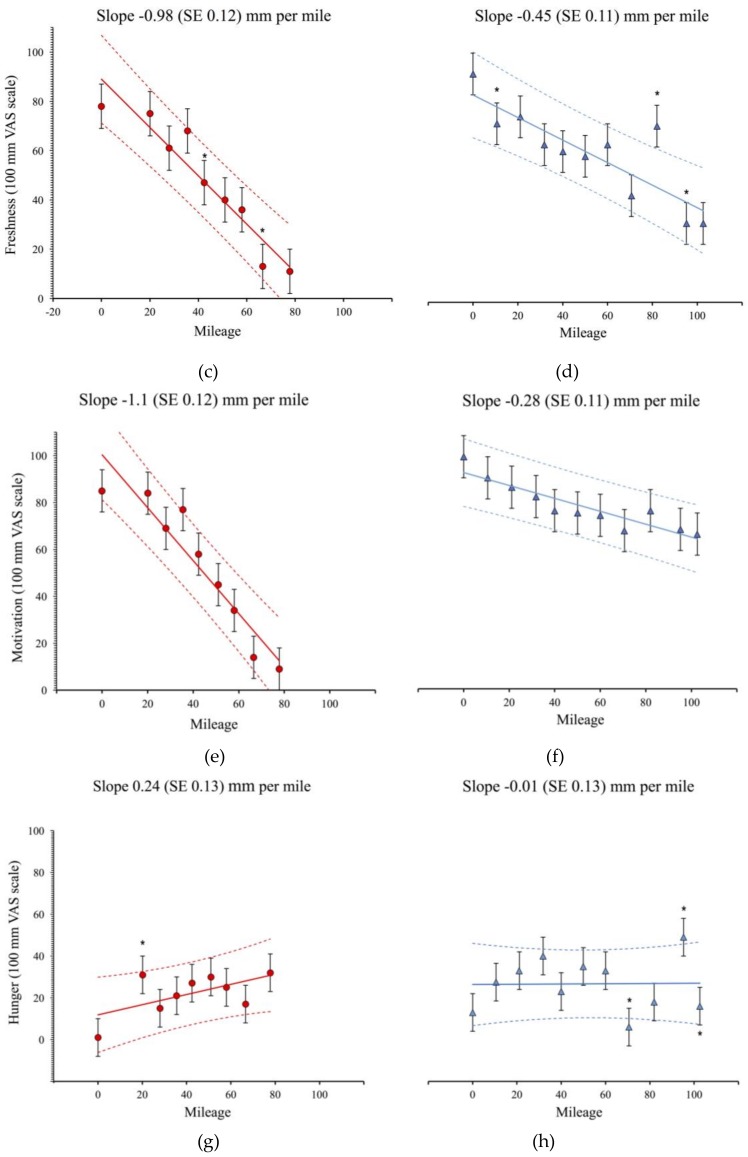

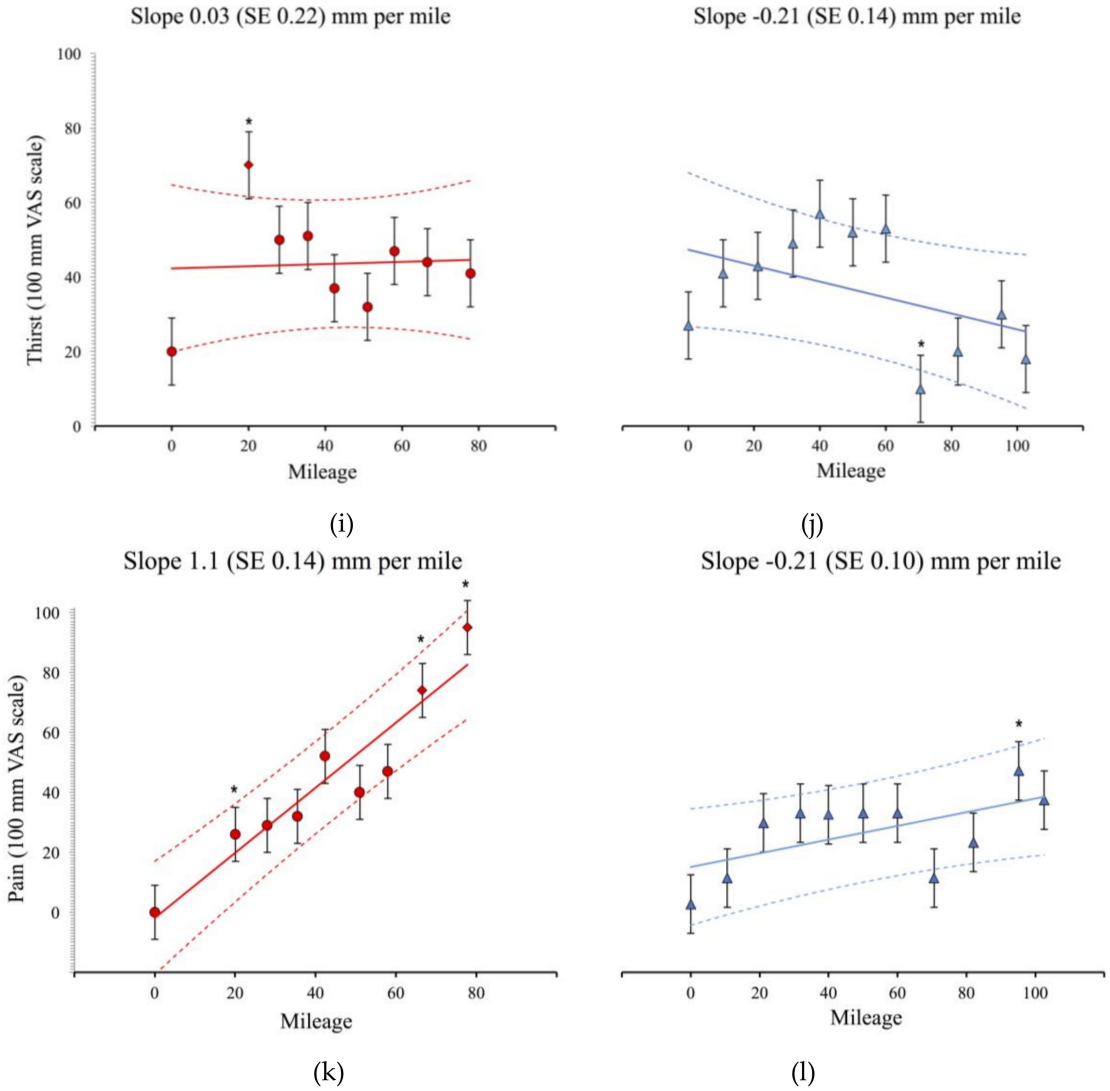
Panels represent the individual data points for race 1 (red circles) and race 2 (blue triangles). Bars represent the typical error of measurement and values falling outside the dashed lines are estimated to be likely (>~75% chance) substantially off the trend [29]. Asterisks (*) represent data points that are estimated to be *likely* different from the previous check point. Panels(**a**) and (**b**) depict RPE trends between events; (**c**) and (**d**) freshness; (**e**) and (**f**) motivation; (**g**) and (**h**) hunger; (**i**) and (**j**) thirst; and (**k**) and (**l**) pain.

The linear trends for all subjective data are presented in Figure 1 and include the slope and standard error. Pearson’s correlations are presented in Table 2. Differences between changes in VAS score were trivial for RPE and thirst, but substantial differences were observed between races in all other subjective measures. Given large to near perfect correlations between pain and freshness and motivation, we chose only to provide detail between race differences for pain only. Therefore, in Table 3 and Table 4, we present a detailed evaluation of the athletes’ perceptions of pain and hunger.

### 3.2. Nutritional Intake

Throughout non-completion, the participant consumed a total of 355.3 g CHO, at a rate of 20.15 g·h^−1^. This equated to a relative consumption of 5.3 g·CHO·kg^−1^. Food frequency data shows the majority of this intake came from real foods and beverages as opposed to specialist sports nutrition products (*n* = 8). Over the course of the completion the participant consumed 592.7 g CHO, at a rate of 25.7 g·h^−1^ (8.8 g.CHO·kg^−1^). Fewer sports nutrition products were also consumed throughout the successful attempt (*n* = 7). Absolute protein intake was 90.1 g and 104 g, resulting in relative intakes of 1.3 g·kg^−1^ and 1.6 g·kg^−1^ for non-completion and completion, respectively. A total intake of 3776 kcal was recorded in completion with 59% or 2246 kcal coming from CHO sources. In contrast 48% or 1347 kcal were obtained from CHO sources during non-completion, with fewer calories being consumed overall (2806 kcal). Principal carbohydrate values were all higher (Glucose 42 g, Fructose 52 g and Sucrose, 98 g) in completion than in non-completion (Glucose 27.3; Fructose 32.2; and, Sucrose 75 g).

## 4. Discussion

The current study describes the assessment and progression of physiological and subjective variables in the context of two contrasting ultra-marathon attempts. These data informed the nutrition, and sports science support strategies that were implemented during and between events. Improvements in subjective measures of pain, motivation, freshness and hunger corresponded to performance resulting in race completion, independent of changes in RPE, which was relatively stable over the course of both attempts. Maximum reported values during both attempts were similar for RPE (non-completion: 4; Somewhat Hard, completion: 5; Hard), whereas other subjective measures oscillated throughout the events, suggesting either that these variables were more sensitive to intervention, or that visual analogue scales provide a more discriminate measure of such variables. Change scores in RPE equated to ~0.5 arbitrary units per 100 miles, hence an alternative scale to the CR-10 [27] is required to detect changes in RPE within this athlete, or over this duration.

No incidence of acute trauma was noted throughout non-completion; as such, the increased pain slope seen in Figure 1 cannot be attributed to an incident, but an inability of the osteoarticular tissue to tolerate periods of sustained running. It is postulated that the Al Andulus trail, completed successfully between the reported attempts served to develop the athlete’s tissue and subjective tolerances for ultra-endurance running. This is supported through regression data suggesting that the best predictors of ultra-marathon performance are mean weekly running kilometres and training speed [10], and recommendations that runners develop strategies to limit tissue damage throughout training and competition [11].

The progression of subjective measures suggests that the strategies to attenuate or modify the perception of pain throughout ultra-running races may also positively impact upon motivation and freshness, and as such improve performance through a combination of increased running speed, fewer walk breaks, and potentially shorter feeding times. Caffeine may be one such strategy; caffeine has been shown to ameliorate leg pain during moderate intensity exercise (60% VO_2max_) in a dose dependent fashion [35], and has been consumed throughout a 100mile race in 3 elite ultra-runners at a rate of 0.9 ± 0.27 mg·kg^−1^·h^−1^ [17]. Timely caffeine consumption may not only alleviate perceptions of pain, but may also improve freshness [36] potentially combatting the circadian challenges of ultra-endurance activity, such as event start time and volume of night time running.

A greater attenuation of hunger as was achieved in race completion, may also be a strategy to indirectly improve freshness and motivation, and may mitigate pain. We observed that hunger was moderately correlated with freshness and motivation, and was largely associated with pain during non-completion (Race 1; Table 2); whereas, during completion when higher nutritional intakes were attained and hunger was perceived to be lower and more stable (Figure 1) correlations between hunger and freshness (Trivial; 0.15, −0.38 to 0.6) and motivation (Trivial; 0.06, −0.45 to 0.54) reduced in magnitude (Race 2; Table 2), with no likely substantial differences in hunger observed during completion (Table 4). This change in hunger, and its ability to ameliorate decreases in freshness and motivation, concomitantly increased the relationship between hunger and pain from large (0.56, −0.04 to 0.86) to very large (0.7, 0.3 to 0.89), suggesting that a well-fed athlete is less likely to report pain, and vice versa, a poorly nourished athlete is more likely to experience pain. The differences in freshness and motivation also suggest that, in this case report, the participant may have differentiated between sensations when hunger was elevated, with freshness, motivation, and pain all perceived discretely and capable of influencing race performance and completion; hence, a negative change in one variable may have produced a further negative cascade between wider measures. Whereas when hunger was lower (completion), pain may have been interpreted as a cumulative integration of sensations of freshness, motivation, and pain.

The higher food and carbohydrate (absolute and relative) consumption achieved during completion supports the notion that trainability of the gut is a contributing factor in successful ultra-endurance exercise performance [16,24]. Events are primarily contested over a fixed distance, and, as such, if an athlete is quicker, the same absolute carbohydrate intake is distributed over a shorter time frame resulting in a higher relative intake. Our data suggest that the athlete managed to improve their tolerance to feeding between attempts, as CHO intake increased by ~5 g·hr^−1^, was tolerated over a longer duration and it formed a larger percentage of nutritional intake (66% increase). Surprisingly, fewer sports nutrition products were consumed throughout the successful attempt. This is contrary to previous reports [17], which suggest that large CHO intakes are readily achieved by using such foodstuffs. Instead, the athlete consumed a variety of CHO sources; preferring fruit at aid stations and rice cakes between stations. This feeding strategy represents current guidance to consume multiple transportable carbohydrates from glucose and fructose sources [23], and is evidenced by the increase across all the constituent sugars between successful and failed attempts. The increase in sucrose consumption from 75 g to 98 g during completion, despite using fewer sports nutrition products, may be an artefact of race duration in that more food was consumed and data obtained.

Mechanistically, the higher CHO intake may have served to combat the decline in running economy and maximal sustainable power likely experienced over the course of the event [12]. A higher CHO intake would elevate the respiratory quotient, improving efficiency and attenuating the expected increase in fractional utilisation of VO_2max_. This may prove advantageous through technical or hilly sections of ultra-endurance events, where an increase in anaerobic energy contribution may be required, and a reciprocal down-regulation of fat metabolism occurs [37,38]. Adopting a low carbohydrate high fat diet is currently in vogue in ultra-endurance sports [37,39]. High rates of fat oxidation (1.54 ± 0.18 g/min) have been observed in trained ultra-distance runners when self-reported habitual dietary changes are implemented over at least one year [39]. A greater percentage fat oxidation would contribute towards sustained energy production in the presence of declining or limited glucose availability, however fat oxidation is not a measure of performance per se. Furthermore, higher fat intakes have been shown to impair higher intensity exercise performance, through a down regulation of CHO metabolism [38,40,41]. In events that are limited by rates of exogenous feeding, and with our data suggesting an improvement (to the point of completion) with greater CHO feeding rates, ‘fat-adaptation’ strategies appear to be impractical and potentially deleterious to the athlete’s performance.

**Figure 2:**
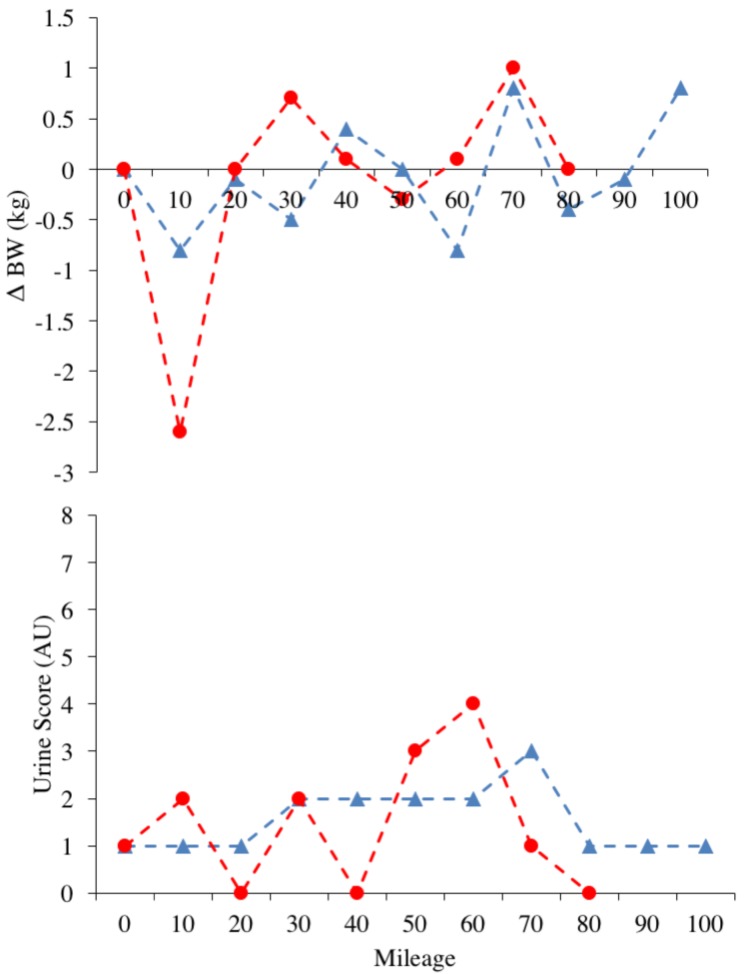
Panels represent the individual data points for race 1 (red circles) and race 2 (blue triangles), for Urine Score and Δ BW.

The role of protein as a substrate has been suggested to increase with exercise duration [42]. Branched chain amino acids are preferentially oxidised and are shown to increase fat oxidation in times of carbohydrate depletion [43]. Due to the catabolic nature and nutritional requirements of ultra-endurance running, branched chain amino acid consumption may attenuate some of the deleterious effects of event participation [43]. Although protein intake increased between events by ~15% (0.3 g·kg^−1^), the difference in protein consumption between attempts is unlikely to have promoted anti-catabolic or oxidative responses because of the concomitant increase in exogenous CHO provision [42]. The extra protein may however have provided a satiating effect i.e., a reduction in hunger (Figure 2).

Hydration needs, as assessed through Δ BW and Urine Score data, were well managed across both events. It is acknowledged that these are indirect measures of hydration, and that both measures are influenced by feeding and substrate usage. However, they present timely and instantaneous methods of obtaining data that can inform strategies in a ‘live’ context [25] and thy have recently been shown to be valid up to −2% Δ BW [44]. Our data suggest that within temperate conditions *ad libitum* drinking can sufficiently maintain indirect hydration measures during ultra-endurance activity of ~24 h in duration. Ad-libitum drinking is favoured throughout ultra-endurance events, and these recommendations are supported by high reported fluid and sodium [8,15] intakes during ultra-marathon running [45] and low incidence of hyponatraemia [19]. Special consideration should be given to individual athletes’ sweat responses and sodium loss through participation in ultra-endurance activity, as very high inter-individual variability in sweat and sodium loss, have been reported [45]. Start time of event may be important in determining these outcomes, as an earlier start time increases the volume of running completed before peak day-time temperatures, serving to decrease sweat rate, as evidenced by a more stable Δ BW (completion start time 6:00 a.m.) and an improved mean Urine Score (non-completion: 4, completion: 3).

Event start time presents a potentially meaningful consideration for future research and support delivery, especially when researchers conduct multiple observations on an athlete(s). Our data suggest differing feeding strategies (and performance) between events of differing start times (10:00 a.m. non-completion; 6:00 a.m. completion). We propose that ‘rate of peak feeding’ i.e., the time at which CHO consumption is highest throughout the event, may be susceptible to either circadian (event start time), duration (time since start), or environmental (altitude, temperature) influences, as all might be drivers of appetite and or exercise performance [46,47,48,49,50]. Exploration into rates of peak feeding may allow for further individualisation and validation of CHO consumption guidelines [22] in ultra-endurance competition, providing scope for more flexible feeding strategies, whilst still meeting energetic demands.

## 5. Conclusions

Pain appears to be a key modulator of ultra-endurance running performance, with improvements in perceptions of pain affecting the sensations of freshness and motivation independent of exercise intensity (RPE). Strategies to modify pain in ultra-endurance athletes might improve performance, although we cannot suggest with certainty whether this is an acquired response (competition/training) or one that is directly mediated by intervention e.g., caffeine. In a moderately trained ultra-endurance athlete, a small improvement in the ability to tolerate CHO feeding resulted in improved performance. Tolerance to CHO feeding is a trainable determinant of ultra-endurance activity with higher rates of CHO positively associated with performance, and training status. Future research should investigate rates of peak feeding throughout similar events, with the goal of ascertaining whether competition nutrition strategies can be tailored specifically towards individual tolerance, within the context of current recommendations, event conditions, and logistics.

## Figures and Tables

**Table 1:** Participant characteristics.

Characteristic	Value
Age	41 years
Height	168 cm
Weight	67.1 kg
Running Experience	5.5 years
Current coaching period	2 years
10 km PB ^1^	39:42
Half Marathon PB	1:26:10
Marathon PB	2:56:49
Ultra-Marathon Experience	Previous DNC ^2^

^1^ PB: Personal Best. ^2^ DNC: Did not complete.

**Table 2:** Pearson’s correlations, 90% confidence intervals and qualitative descriptors for VAS measures. DNC: did not complete.

RACE 1 (DNC)	Freshness	Motivation	Hunger	Thirst	Pain
Freshness	1.00	Near Perfect (0.99, 0.96 to 1.0)	Moderate (0.40, −0.24 to 0.80)	Small (0.14, −0.49 to 0.67)	Near perfect (0.93, 0.76 to 0.98)
Motivation	–	1.00	Moderate (0.38, −0.26 to 0.79)	Small (0.14, −0.49 to 0.67)	Near perfect (0.91, 0.69 to 0.98)
Hunger	–	–	1.00	Small (0.48, −1.5 to 0.83)	Large (0.56, −0.04 to 0.86)
Thirst	–	–	–	1.00	Trivial (0.08, −0.53 to 0.64)
Pain	–	–	–	–	1.00
**RACE 2**	**Freshness**	**Motivation**	**Hunger**	**Thirst**	**Pain**
Freshness	1.00	Near Perfect (0.90, 0.74 to 0.97)	Trivial (0.15, −0.38 to 0.6)	Moderate (0.31, −0.22 to 0.7)	Large (0.62, 0.17 to 0.85)
Motivation	–	1.00	Trivial (0.06, −0.45 to 0.54)	Small (0.24, −0.29 to 0.66)	Large (0.63, 0.19 to 0.86)
Hunger	–	–	1.00	Large (0.6, 0.14 to 0.85)	Very large (0.7, 0.3 to 0.89)
Thirst	–	–	–	1.00	Moderate (0.33, −0.2 to 0.71)
Pain	–	–	–	–	1.00

**Table 3:** Increases in perceived pain over each ultra-marathon run including the likelihood that the athlete was experiences a substantial increase in pain. Likelihoods are presented with the qualitative inference and percentage chance that the increase was substantial or trivial. Differences between are presented as raw change and 90% confidence interval, with the qualitative inference.

Miles (x)	Increases in Perceived Pain per x Miles (mm, 90% CI)	Was the Athlete Experiencing a Substantial Increase in Pain?	Increases in Perceived Pain per x Miles (mm, 90% CI)	Was the Athlete Experiencing a Substantial Increase in Pain?	Difference in Increase of Perceived Pain in Race 2. (mm, 90% CI)
10	9.3, 7.5 to 11	Unlikely (6/94%)	2.1, 0.5 to 3.7	Most unlikely (100/0%)	Most likely, trivial (7.2, 4.9 to 9.5)
20	22, 20 to 24	Very likely (99/1%)	4.2, 2.6 to 5.9	Most unlikely (100/0%)	Most likely, less painful (18, 15 to 20)
40	43, 42 to 45	Almost certainly (100/0%)	8.4, 6.8 to 10	Unlikely (19/81%)	Most likely, less painful (35, 33 to 37)
60	65, 63 to 67	Almost certainly (100/0%)	13, 11 to 14	Possibly (54/46%)	Most likely, less painful (52.5, 50.2 to 54.8)
80	87, 85 to 89	Almost certainly (100/0%)	17, 15 to 19	Possibly (73/27%)	Most likely, less painful (70, 68 to 72)

The point at which the increases in perceived pain was *likely* substantial was ~15 miles in race 1 and ~100 miles in race 2.

**Table 4:** Increases in perceived hunger over each ultra-marathon run including the likelihood that the athlete was experiencing a substantial increase in hunger. Likelihoods are presented with the qualitative inference and percentage chance the athlete was substantially more hungry, trivial, or less hungry. Differences between are presented as raw change and 90% confidence interval, with the qualitative inference.

Miles (x)	Change in Hunger per x Miles (mm, 90% CI)	Was the Athlete Experiencing a Substantial Increase in hunger?	Increases in Perceived Hunger per x Miles (mm, 90% CI)	Was the Athlete Experiencing a Substantial Increase in Hunger?	Difference in Increase of Perceived Hunger in Race 2. (mm, 90% CI)
10	2.4, 0.3 to 4.6	Most unlikely (0/100/0%)	0.1, −2.1 to 2.2	Most unlikely (0/100/0%)	Most likely, trivial (2.4, -0.5 to 5.3)
20	4.9, 2.7 to 7.0	Very unlikely (1/99/0%)	0.1, −2.0 to 2.3	Most unlikely (0/100/0%)	Most likely, trivial (4.8, 1.9 to 7.6)
40	9.7, 7.6 to 12	Possibly (33/67/0%)	0.2, −1.9 to 2.4	Very unlikely (2/96/2%)	Likely trivial (9.5, 6.6 to 12)
60	15, 13 to 17	Possibly (63/36/0%)	0.4, −1.8 to 2.5	Unlikely (8/86/7%)	Likely, lower ↑ hunger (14, 11 to 17)
80	20, 17 to 22	Likely (76/23/1%)	0.5, −1.7 to 2.6	Unlikely (14/74/12%)	Most likely, lower ↑ hunger (19, 16 to -22)

The point at which the increase in perceived hunger was *likely* substantial was ~80 miles in race 1.
